# Polymerized injectable platelet-rich fibrin with octacalcium phosphate (bontree®) for treatment of periodontal intrabony defects assessed using CBCT: A randomized controlled clinical trial

**DOI:** 10.6026/973206300222718

**Published:** 2026-05-31

**Authors:** P.P Safwana, Sreekanth Puthalath, Deepak Thomas, Deepu Mathews Panickal, N Shahna, Maria Thomas

**Affiliations:** 1 Department of Periodontics and Implantology, Educare Institute of Dental Sciences, Malappuram, Kerala, India

**Keywords:** Injectable platelet-rich fibrin, octacalcium phosphate (OCP), cone-beam computed tomography (CBCT), periodontal regeneration

## Abstract

Periodontal intrabony defects remain a significant clinical challenge due to the difficulty in achieving predictable regeneration of lost alveolar bone. Therefore, it is of interest to evaluate the effectiveness of injectable platelet-rich fibrin (I-PRF) polymerized with octacalcium phosphate (OCP) compared to OCP alone in treating intrabony defects using a split-mouth design. Hence, twelve patients with chronic periodontitis contributed twenty-four sites, assessed clinically and radiographically using CBCT at baseline and six months. Both groups demonstrated significant improvements in clinical and radiographic parameters, with greater percentage changes observed in the I-PRF + OCP group, although intergroup differences were not statistically significant. The adjunctive use of polymerized I-PRF with OCP shows a favorable trend in enhancing periodontal regeneration and warrants further investigation in larger studies.

## Background:

Periodontitis is a host-microbe driven inflammatory disease that gradually disintegrates the tooth-supporting apparatus, comprising of alveolar bone, periodontal ligament and cementum [1]. Dissemination of bacterial toxins across the epithelial barrier also results in cascade of immunological responses, which eventually leads to the destruction of tissues and the loss of clinical attachments [2]. Therefore, the restoration of periodontal structures that have been lost has been a clinical goal of periodontal therapy where a complex interaction between biological mediators, proper populations of cells and the proper scaffold materials is needed [3]. It has been established over the last few decades that a great variety of bone graft materials have been produced and perfected in the treatment of periodontal bone defects beginning with autogenous grafts, continuing in the line of synthetic alloplastic grafts [4]. Octacalcium phosphate (OCP) is one of the calcium phosphate biomaterials which are promising as a synthetic bone substitute due to its biodegradability, neutral PH and high osteoconductive qualities [5]. The idea that the biological apatite is found in the bones and teeth as crystals of OCP were initially hypothesized several decades ago, which has defined a solid background of its use in the clinical sphere [6]. Further researches have validated that synthetic OCP has a superior osteoconductivity to conventional calcium phosphate ceramics, such as non-sintered hydroxyapatite, owing in part to a structural transformation to the apatite phase [7]. Grafts like Bontree 10 which is commercially available as OCPs have proven to be highly osteogenic and fast bio absorbable in preclinical and clinical studies [8]. Along with improvements in scaffold materials, autologous platelet concentrates have changed the regenerative periodontal therapy. Platelet-rich fibrin (PRF), which falls into the category of second-generation platelet concentrates, is an assortment of three-dimensional fibrin matrix that delivers gradual polymerization and prolonged discharge of development factors and cytokines through wound healing phases [9]. A major advancement was the creation of injectable platelet-rich fibrin (I-PRF) using altered low-speed centrifugation regimens, which could be handled in liquid phase, provided better distribution of growth factors and was capable of being polymerized with particulate graft substances [10]. It has been shown that I-PRF releases much more amounts of various growth factors than previous platelet concentrate preparations [11]. Moreover, comparative studies have found out that I-PRF induces much higher osteoblast differentiation and proliferation compared to platelet-rich plasma [12]. Clinical PRF use in periodontal intrabony defects has provided positive results separately and when combined with bone grafts and it has also shown an increase in clinical attachment gain and radiographic bone fill improvements [13, 14]. Other studies that use drugs with PRF have had higher therapeutic responses that highlight the even more adaptable nature of this biomaterial [15, 16]. Therefore, it is of interest to assess the clinical and radiographic effectiveness of I-PRF which was polymerized with octacalcium phosphate bone graft in treating periodontal intrabony defects through the use of cone-beam computed tomography.

## Materials and Methods:

## Design of the study and ethical considerations:

It was a randomized controlled clinical trial using split-mouth design and was done on patients who visited outpatient department of a dental institution after receiving the approval of the institutional ethical committee in accordance with the Declaration of Helsinki (1964, revised 2013).

## Sample size and subject selection:

The study enrolled twelve patients who presented with chronic periodontitis each contributing a total of twenty-four locations of intrabony defects. The sample size was calculated using the past research on the suitability of bone graft material in intrabony defects with the power of 80% and a significance of 0.05. The inclusion criteria included: Age of 25 years to 60 years; either gender; good systemic health and no contraindication to periodontal surgery or local anesthesia; cooperative subjects with the commitment to maintain their oral health; and minimum two intrabony defects; one in each quadrant or contralateral side of the same arch; with radiographic evidence of vertical or angular bone loss. The exclusion criteria were: having a relevant system or disorder that might interfere with wound healing; pregnant or lactating; undergoing periodontal treatment within the previous six months; having an adverse habit such as smoking, chewing tobacco or alcohol; known allergy or hypersensitivity to study materials; physical/mental disability preventing informed participation.

## Group allocation:

The split-mouth design was used to randomly assign sites to two groups. Group A (Control): Intrabony defects opened up with open flap debridement and octacalcium phosphate bone graft (Bontree 4, HudensBio Co., Gwangju, Korea) alone. Group B (Test): Intrabony defects and then treated with open flap debridement and injectable platelet-rich fibrin polymerized with octacalcium phosphate bone graft.

## Injectable Platelet-Rich Fibrin Preparation Injectable platelet-rich fibrin is prepared through several steps:

Firstly, platelet donors are collected and stored under refrigeration until required. Injectable platelet-rich fibrin is made by a series of steps: Firstly, platelet donors are frozen and kept in refrigeration until the requirement. The additive-free dry vacutainer tubes were filled with venous blood of the antecubital fossa. The tubes were then put down quickly in a horizontal centrifugation at 3300 rpm in two minutes with a balance tube containing water. After centrifugation a layer of I-PRF was removed in orange color with an 18-gauge needle without bringing about homogenization of the layered components. Pre-operative Protocol Every patient received extensive phase I treatment comprising of scaling and root planing. After a re-evaluation phase (satisfactory plaque control was observed), baseline clinical parameters and CBCT imaging were determined. To have consistent clinical measurements, custom acrylic occlusals that were made on the casts of the study were used as fixed reference points. Clinical parameters at the baseline and 6 months of post-operation, the following parameters were measured

## Using UNC-15 periodontal probe:

[1] Gingival Index (GI) - Loe and Silness (1963)

[2] Probing Pocket Depth (PPD) - converted into gingival margin to the base of pocket.

[3] Relative Attachment Level (RAL) - between the custom occlusal stent and the pocket base.

## Surgical procedure:

Full-thickness mucoperiosteal flaps were reflected under local anesthesia to reveal the base of each of the osseous defects. Total debridement and root planing were done on all sites. In Group A sites, OCP bone graft was collimated in the defect. The aspirated I-PRF was put in a sterile metal container in Group B sites and OCP granules were added slowly. The obtained I-PRF-OCP complex was after about twenty minutes of polymerization placed and condensed into the defect. All the flaps were re-positioned and stabilized using 3-0 silk sutures after which periodontal dressing was applied.

## Radiographic assessment:

The baseline and six months post-operative CBCT scans were obtained. The measurements were made and obtained as follows:

[1] Height of defect (CEJ-BD): Cemento-enamel junction to bottom of defect.

[2] Impression: cemento-enamel junction to alveolar crest.

[3] Depth of defect (AC-BD): Crest alveolar to base of defect.

[4] Defect mesiodistal (MD) and buccolingual (BL) width.

## Statistical analysis:

The SPSS version 26.0 (SPSS Inc., Chicago, IL) was used to analyze the data. The Shapiro-Wilk test was used to test normality. The paired sample t-test was used to undertake intragroup comparisons whereas independent sample t-test was used to undertake intergroup comparisons. The significance level was established as p <0.05.

## Results:

All twelve patients completed the six-month follow-up without significant post-operative complications. Healing progressed uneventfully at all twenty-four surgical sites. Both groups demonstrated statistically significant improvements in all clinical parameters from baseline to six months (p = 0.0001 for all within-group comparisons). The percentage reductions were consistently greater in Group B compared to Group A across all clinical parameters: plaque index (42.7% vs. 40.4%), gingival index (40.9% vs. 33.2%), probing pocket depth (62.5% vs. 56.4%) and relative attachment level (42.7% vs. 39.6%). However, intergroup differences did not reach statistical significance at any evaluation interval (Table 1). CBCT analysis revealed statistically significant reductions in defect height (CEJ-BD), defect depth (AC-BD), mesiodistal width and buccolingual width within both groups from baseline to six months (Table 2). The alveolar crest level (CEJ-AC) did not demonstrate statistically significant changes in either group (Group A: p = 0.42; Group B: p = 0.25). Group B demonstrated greater percentage changes in all radiographic parameters, particularly in defect depth (66.2% vs. 59.5%) and mesiodistal width (25.8% vs. 17.3%). No statistically significant intergroup differences were observed (Table 3).

## Discussion:

Results of the current research indicate that octacalcium phosphate as single molecule and OCP polymerized with injectable platelet-rich fibrin are effective treatment modalities of periodontal intrabony defects that lead to significant clinical and radiographic changes within a six-month period of observation. Although the improvement percentage was higher in the adjunctive use of I-PRF in all the parameters measures, the intergroup differences were not statistically significant, which was probably influenced by the small sample size of the preliminary study. The substantial within-group changes in plaque and gingivitance indices of the two groups indicate the successful oral hygiene maintenance program and Phase 1 Therapy which were used before the surgical intervention. Such results are congruent with other studies that have shown that meticulous plaque control is the basis of successful regenerative outcome irrespective of the biomaterial that is used [17]. The similarity of the intergroup outcomes of the soft tissue indices indicates that neither of the treatment modalities affects the supragingival plaque accumulation or gingival inflammation independently since the parameters are largely determined by the patient compliance and home care routines [18]. These significant decreases in probing pocket depth with Group B nearing statistical significance in the intergroup comparison (p = 0.06) are consistent with the available evidence on the regenerative qualities of calcium phosphate-based bone substitutes in periodontal defects [19]. The new bone formation is supported by the osteoconductive properties of octacalcium phosphate based on its own structural transition mechanism, according to which OCP is a precursor of biological apatite that is gradually transformed into hydroxyapatite but retains the three-dimensional architecture of the scaffold required to support the migration of cells and vascular ingrowth [20]. This extra effect of the I-PRF polymerization can be explained by the fact that the platelet-derived growth factor, transform growth factor- beta and vascular endothelial growth factor continue to leak out of the fibrin matrix that facilitates angiogenesis, cell growth and osteoblast differentiation at the area of injury [21]. CBCT analysis radiographic results were good three-dimensional evaluations of the defect healed results which indicated significant bone fill on both treatment groups. Notably, the lack of meaningful difference in the alveolar crest height (CEJ-AC) of the two groups is notable and is in line with other studies that have noted that crestal bone remodeling can be very slow and may take a longer duration to produce changes that are detectable [22].

Studies that used bovine porous bone mineral with guided tissue regeneration have also shown minimal crestal bone response after six months in spite of massive progress on intrabony defect fill [23]. The higher percentage decrease in defect depth and width size in Group B is evidence of the biological explanation of the combination of growth factor-rich preparations and the osteoconductive scaffold. This result compares to reports of other studies involving the use of different types of platelet concentrates together with bone substitutes whereby increase in defect repair was linked to synergetic interaction between the scaffold architecture and biological mediators [24, 25]. Polymerized complex of I-PRF-OCP complex has its own clinical benefits, which are independent of the biological synergy of its constituents. Polymerization process forms a cohesive moldable mass that can be accurately fitted on an irregular morphology of intrabony defect to enhance graft stability and minimize the chances of particle displacement during repositioning of flaps and suture. This enhanced manipulation property is a critical pragmatic benefit compared to the positioning of the loose particulate graft material that may be susceptible to dislocation especially in non-contained defect morphologies [26]. Other clinical trials that have been previously conducted to assess platelet concentrates using different bone substitutes have shown mixed outcomes as far as the statistical significance of intergroup comparison is concerned. The use of PRF with xenografts and bioactive glass has shown tendencies towards the use of the combination therapy without always achieving statistical significance [27, 28]. Conversely, studies that have greater numbers of participants and extended follow-ups have sometimes shown that the combination therapy had huge benefits especially in radiographical defect fill and clinical attachment gain [29, 30]. The current results are well aligned with this larger body of evidence, where in albeit that there is a statistically significant clinical value of the adjunctive use of I-PRF, multicenter trials of adequate power are required to confirm beyond reasonable doubt the statistical significance of the combination method. The shortcomings of the research are in the limited sample size, six months follow-up which might not be long enough to determine the full magnitude of bone remodeling, as well as lack of open flap debridement-only control group. Also, no histological analysis of the regenerated tissues was done, which ruled out conclusive studies on the nature of healing- whether regeneration with the formation of new attachments was achieved or that it was simply a defect fill by a repair tissue. Further research ought to overcome these shortcomings and also examine the possibilities of this combination therapy in more demanding clinical situations like furcation defects and non-contained defect morphology.

## Conclusion:

We show that octacalcium phosphate bone graft, individually and with injectable platelet-rich fibrin, showed significant clinical and radiographic outcome over six months in the treatment of periodontal intrabony defects. To the best of the evaluation of all parameters, polymerized I-PRF and OCP combination had a higher percent improvement than OCP alone, but the difference between the intergroup was not statistically significant. The graft material was improved in the number of handling characteristics and defect adaptation due to the polymerization process. Such results indicate that injectable platelet-rich fibrin with octacalcium phosphate bone graft has potential as a regenerative modality, which can be further studied in larger, longitudinal, multicenter studies requiring histology to verify its results.

## Figures and Tables

**Table 1:** Comparison of clinical parameters within and between groups

**ESBL Gene**	**n (%)**	**Most Common Variant**	**Variant n (%)**
blaCTX-M (total)	153 (82.3%)	-	-
CTX-M Group 1	132 (71.0%)	blaCTX-M-15	118 (63.4%)
CTX-M Group 9	18 (9.7%)	blaCTX-M-14	14 (7.5%)
CTX-M Group 2	3 (1.6%)	blaCTX-M-2	3 (1.6%)
blaTEM (total)	104 (55.9%)	blaTEM-1 (non-ESBL)	86 (46.2%)
		blaTEM-52	10 (5.4%)
		blaTEM-30	8 (4.3%)
blaSHV (total)	48 (25.8%)	blaSHV-12	22 (11.8%)
		blaSHV-11 (non-ESBL)	16 (8.6%)
		blaSHV-5	10 (5.4%)
blaOXA-1-like	62 (33.3%)	blaOXA-1	58 (31.2%)
Co-carriage patterns			
CTX-M + TEM	52 (28.0%)	-	-
CTX-M + OXA	38 (20.4%)	-	-
CTX-M + TEM + OXA	28 (15.1%)	-	-
CTX-M + SHV	24 (12.9%)	-	-
CTX-M + TEM + SHV + OXA	12 (6.5%)	-	-
*Statistically
significant (p < 0.05)

**Table 2:** Comparison of CBCT parameters within and between groups

**Sequence Type**	**n (%)**	**Phylogenetic Group**	**Predominant ESBL Gene**	**Fluoroquinolone Resistance n (%)**	**Specimen Source (Most Common)**
ST131	46 (24.7%)	B2	blaCTX-M-15 (89.1%)	43 (93.5%)	Blood (34.8%), Urine (32.6%)
ST405	22 (11.8%)	D	blaCTX-M-15 (72.7%)	18 (81.8%)	Urine (40.9%), Respiratory (27.3%)
ST648	16 (8.6%)	F	blaCTX-M-15 (62.5%)	14 (87.5%)	Urine (37.5%), Wound (25.0%)
ST38	12 (6.5%)	D	blaCTX-M-14 (50.0%)	7 (58.3%)	Urine (50.0%), Blood (25.0%)
ST410	10 (5.4%)	A	blaCTX-M-15 (80.0%)	8 (80.0%)	Respiratory (40.0%), Blood (30.0%)
ST69	8 (4.3%)	D	blaCTX-M-15 (62.5%)	4 (50.0%)	Urine (62.5%), Other (25.0%)
Others (22 STs)	72 (38.7%)	Mixed	Mixed	52 (72.2%)	Mixed
*Statistically
significant (p < 0.05)

**Table 3:** Comparison of defect width parameters within and between groups

**Antimicrobial Agent**	**Susceptible n (%)**	**Intermediate n (%)**	**Resistant n (%)**
Ampicillin	0 (0%)	0 (0%)	186 (100%)
Amoxicillin-clavulanate	68 (36.6%)	46 (24.7%)	72 (38.7%)
Piperacillin-tazobactam	128 (68.8%)	26 (14.0%)	32 (17.2%)
Cefotaxime	0 (0%)	4 (2.2%)	182 (97.8%)
Ceftazidime	14 (7.5%)	12 (6.5%)	160 (86.0%)
Cefepime	42 (22.6%)	28 (15.1%)	116 (62.4%)
Aztreonam	18 (9.7%)	16 (8.6%)	152 (81.7%)
Imipenem	178 (95.7%)	2 (1.1%)	6 (3.2%)
Meropenem	179 (96.2%)	1 (0.5%)	6 (3.2%)
Ertapenem	176 (94.6%)	3 (1.6%)	7 (3.8%)
Ciprofloxacin	38 (20.4%)	2 (1.1%)	146 (78.5%)
Levofloxacin	44 (23.7%)	4 (2.2%)	138 (74.2%)
Gentamicin	80 (43.0%)	4 (2.2%)	102 (54.8%)
Amikacin	163 (87.6%)	8 (4.3%)	15 (8.1%)
Tobramycin	66 (35.5%)	6 (3.2%)	114 (61.3%)
Trimethoprim-sulfamethoxazole	32 (17.2%)	2 (1.1%)	152 (81.7%)
Nitrofurantoin	157 (84.4%)	12 (6.5%)	17 (9.1%)
Fosfomycin	173 (93.0%)	4 (2.2%)	9 (4.8%)
Tigecycline	179 (96.2%)	4 (2.2%)	3 (1.6%)
Colistin	185 (99.5%)	0 (0%)	1 (0.5%)
*Statistically
significant (p < 0.05)

## References

[1] Chaudhary B (2023). J Indian Soc Periodontol..

[2] Rithesh K (2025). J Indian Soc Periodontol..

[3] Alshoiby MM (2023). Clin Oral Investig..

[4] Baghele O (2023). Quintessence Int..

[5] Salem S (2023). Natl J Maxillofac Surg..

[6] Atchuta A (2020). J Indian Soc Periodontol..

[7] Bahammam MA (2021). Saudi J Biol Sci..

[8] Mallappa J (2022). J Indian Soc Periodontol..

[9] Miron RJ (2025). Periodontol 2000..

[10] Gummaluri SS (2020). J Dent Res Dent Clin Dent Prospects..

[11] Thakkar B (2020). J Int Soc Prev Community Dent..

[12] Paolantonio M (2020). J Periodontol..

[13] Nair UP (2022). Bioengineering (Basel)..

[14] Gautam K (2022). Contemp Clin Dent..

[15] Sharma P (2023). J Indian Soc Periodontol..

[16] Pham TAV. (2021). J Evid Based Dent Pract..

[17] Elbehwashy MT (2021). Clin Oral Investig..

[18] Miron RJ (2021). Clin Oral Investig..

[19] Lei L (2020). J Periodontol..

[20] Csifó-Nagy BK (2021). BMC Oral Health..

[21] El Soudany KS (2023). J Oral Maxillofac Pathol..

[22] Shadap NKS (2023). Int J Clin Pediatr Dent..

[23] Abdulrahman YA (2022). Clin Oral Investig..

[24] Deepika M (2023). J Contemp Dent Pract..

[25] Mamajiwala AS (2021). Clin Oral Investig..

[26] Hazari V (2021). Contemp Clin Dent..

[27] Ustaoglu G (2020). Med Oral Patol Oral Cir Bucal..

[28] Rexhepi I (2021). J Periodontol..

[29] Liu K (2021). J Periodontol..

[30] Zhou Z (2020). J Oral Biol Craniofac Res..

